# Risk Factors, Genetic Diversity, and Antimicrobial Resistance of *Staphylococcus* spp. Isolates in Dogs Admitted to an Intensive Care Unit of a Veterinary Hospital

**DOI:** 10.3390/antibiotics12030621

**Published:** 2023-03-21

**Authors:** Jordana Almeida Santana, Amanda Oliveira Paraguassu, Ranielle Stephanie Toledo Santana, Rafael Gariglio Clark Xavier, Patricia Maria Colleto Freitas, Flavia Figueira Aburjaile, Vasco Ariston de Carvalho Azevedo, Bertram Brenig, Anders Miki Bojesen, Rodrigo Otávio Silveira Silva

**Affiliations:** 1 Departamento de Medicina Veterinária Preventiva, Universidade Federal de Minas Gerais (UFMG), Belo Horizonte 30720440, Brazil; 2 Instituto de Ciências Biológicas (ICB), Universidade Federal de Minas Gerais (UFMG), Belo Horizonte 30720440, Brazil; 3 Institute of Veterinary Medicine, University of Göttingen, 37077 Göttingen, Germany; 4 Department of Veterinary and Animal Sciences, Faculty of Health and Medical Sciences, University of Copenhagen, 2820 Copenhagen, Denmark

**Keywords:** methicillin resistance, *S. pseudintermedius*, *S. aureus*, nosocomial transmission, clonal dispersal

## Abstract

Intensive Care Units (ICU) usually provide an excellent environment for the selection of pathogens associated with hospital-acquired infections (HAI), leading to increased mortality and hospitalization costs. Methicillin-resistant *Staphylococcus pseudintermedius* (MRSP) is a major cause of HAI in dogs worldwide, but the risk factors and dynamics of colonization by MRSP are largely unknown. This study aimed to evaluate the risk factors associated with the acquisition of MRSP in dogs admitted to an ICU, and to report the antimicrobial resistance profiles and genetic relatedness of MRSP isolates. Sterile swabs from the nostril, axilla, and rectum were collected daily during the hospitalization of 54 dogs. Samples were subjected to Mannitol Salt Agar, and colonies were identified by MALDI-ToF, polymerase chain reaction (PCR), and sequencing of the *rpoB* gene. Antimicrobial susceptibility testing and PCR detection of *mecA* were performed. *Staphylococcus* spp. was isolated from 94% of the dogs, and the most frequently isolated species was *S. pseudintermedius* (88.2%). Carriage of multidrug resistant (MDR) staphylococci was observed in 64.4% of the dogs, and approximately 39% had methicillin-resistant *Staphylococcus* sp. (MRS), of which 21.6% had MRSP and 1.9% had methicillin-resistant *S. aureus* (MRSA). The acquisition of MRSP during ICU hospitalization was associated with sex (female), age (>7 years), and dogs that had previously been treated with antimicrobials. Animals colonized by MRSP resistant to ≥9 antimicrobial classes had longer hospital stays than those colonized by other MRS strains. Among the 13 MRSP isolates that were subjected to whole-genome sequencing, ten were classified as ST71. A single nucleotide polymorphism (SNP) analysis revealed three clones, including one that was detected in infected dogs outside the ICU. This study indicates novel risk factors associated with colonization by MRSP. The detection of the same MRSP clone causing HAI outside the ICU reinforces the need for improved infection prevention and control practices at veterinary hospitals in general and at the ICU in particular.

## 1. Introduction

In recent decades, many technical advances have been made in veterinary medicine to improve and prolong the life of animals. However, invasive surgical procedures, immunosuppressive therapies, antimicrobials, and prolonged hospitalizations, including in Intensive Care Units (ICU), have contributed to the increase in hospital-acquired infections (HAI) and the spread of multidrug-resistant pathogens in veterinary hospitals [1]. These pathogens are associated with increased mortality and economic burden due to the costs associated with extended hospital stays [2]. Several staphylococci have been associated with HAI in humans and animals, including *S. aureus, S. haemolyticus* and other species. In dogs, methicillin-resistant *S. pseudintermedius* (MRSP) has been described as a frequent cause of community-acquired HAI in dogs worldwide [3,4,5,6,7,8,9]. Although often isolated from outpatients, this bacterium has also been isolated from the environment, including surfaces, equipment, and fomites, as well as from staff and inpatients [3,9,10,11]. Although infrequent, MRSP has also been reported to infect humans [12,13].

Despite its importance, the dynamics of MRSP colonization in the hospital environment remain poorly understood. In this context, ICU stands out as an environment that provides favorable selection conditions and survival for multidrug-resistant pathogens [9,14]. A review of the clinical records of inpatients at the Veterinary Hospital of the Federal University of Minas Gerais (HV-UFMG) in the last four years indicated MRSP as one of the main causes of HAI at the institution. Later, a study in the same hospital revealed an infection by MRSP from nine novel sequence types (ST), and also by ST71 [15], an ST already reported in a large HAI outbreak elsewhere [6,15]. Together, these findings raised the need for a better understanding of the presence and transmission of antimicrobial resistant staphylococci, particularly MRSP, in the hospital facilities. Thus, the present study aimed to evaluate staphylococcal colonization and the risk factors associated with the acquisition of antimicrobial resistant staphylococci in dogs admitted to an ICU, and to report the antimicrobial resistance profiles and genetic relatedness of the isolates.

## 2. Results

### 2.1. Data Collection

A total of 501 nasal, rectal, and axillary swabs were collected from 54 dogs admitted to the ICU, resulting in 211 (42.1%—211/501) *Staphylococcus* spp. isolates from 51 animals (94.4%—51/54). The clinical and epidemiological variables of the dogs included in this study are described in Table 1. The duration of ICU hospitalization ranged from one to ten days, with a mean of three days (±2.1). The animals belonged to different breeds and ages, ranging from 15 days to 18 years old, with a mean age of 88 months (±61.4). The majority of the dogs were female (64.1%—34/53), had contact with other animals in their household (69.8%—30/43), and received antimicrobial treatment during their stay in the ICU (77.3%—41/53). Approximately half of the dogs were classified as elderly (50%—23/46) and were administered antimicrobials prior to ICU admission (48.8%—20/41). Twenty animals died during hospitalization (37.7%—20/53). More detailed data for each individual participating dog are summarized in Supplementary File S1.

### 2.2. Staphylococcus spp. Isolation and Identification

Two hundred and eleven staphylococcal isolates were obtained from 501 swab samples originating from fifty-one dogs admitted to the ICU of HV-UFMG. Descriptions of hospitalized dogs, positive dogs, *Staphylococcus* spp., *S. pseudintermedius* and MRSP isolates recovered per day are summarized in Table 2.

Of the 211 isolates of *Staphylococcus* spp., 85 (50.9%, 85/167), 72 (43.1%, 72/167), and 54 (32.3%, 54/167) were recovered from the nasal, rectal, and axillary sites, respectively (Table 2). There was a difference in the frequency of staphylococcal isolation between the sample sites (*p* = 0.05), with the nasal site showing a similar isolation frequency to the rectal site (*p* = 0.226), which was higher than that at the axillary site (*p* = 0.001); the frequency of isolation at the rectal site was statistically equal to that at the axillary site (*p* = 0.07) (Table 3).

Twelve species of *Staphylococcus* were identified among the 211 isolates obtained (Table 3). *S. pseudintermedius* was the most frequent (71.1%—150/211), and was at least six times more likely to be isolated than any other species identified in this study (*p* < 0.001). *S. pseudintermedius* was recovered from 45 dogs (45/54—83.3%) and showed a statistically higher isolation rate at the nasal (39.3%, *p* = 0.002) and rectal (38.0%, *p* = 0.005) sites than at the axillary site (22.7%).

*S. aureus* was the second most common species (12.8%); however, with the exception of *S. pseudintermedius*, there was no statistical difference between the frequency of isolation and the sum of the frequencies of other agents (*p* = 0.4861). This bacterium was isolated from 13 dogs (24.1%, 13/54), mainly at the nasal site. In fact, *S. aureus* was 5.6 times more likely to be isolated from the nasal site than the sum of the other agents, except for *S. pseudintermedius* (*p* = 0.005).

In addition to *S. pseudintermedius* and *S. aureus*, the other *Staphylococcus* species identified in the present study (n = 10) were isolated at a frequency below 7% (Table 3). Regarding the diversity of staphylococcal species in each sampled site, 11 out of the 12 species were detected in the axillary site, resulting in a higher diversity than that in the nasal site (41.6%, *p* = 0.027).

### 2.3. Antimicrobial Susceptibility

**Figure 1:**
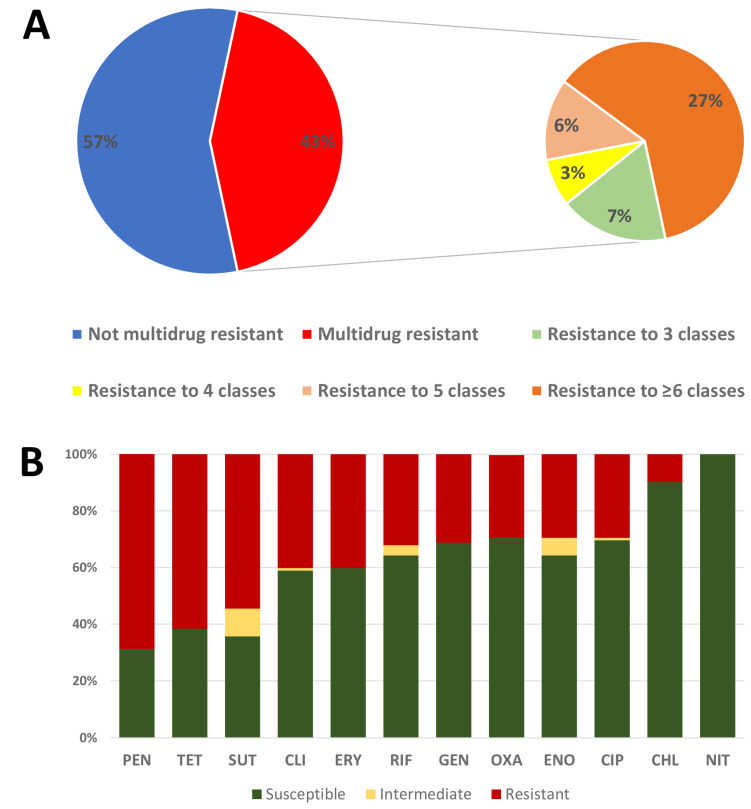
(**A**) Total frequency (%) of multidrug resistant (MDR) staphylococci. (**B**) Percentage of *S. pseudintermedius* isolates with antimicrobial resistance recovered from dogs admitted to the ICU at HV-UFMG between August 2018 and April 2019.

Of the 211 isolates, 158 (74.9%) were resistant to at least one of the antimicrobials tested, and 92 (43.6%) were multidrug-resistant (Figure 1A). Among the dogs that were positive for *Staphylococcus* spp., 64.7% (33/51) carried multidrug-resistant strains and 39.2% (20/51) carried MRS isolates at least once during the study (Supplementary File S1). Penicillin G was the antimicrobial with the greatest number of resistant isolates (57.8%, 122/211) (Supplementary File S1), being statistically equal to that of tetracycline (52.1%, 110/211) (*p* = 0.281) but higher than those of the others (*p* = 0.008). The resistance rate to most antimicrobials was between 20 and 50%, with the exception of chloramphenicol (7.6%, 16/211) and nitrofurantoin (1.9%, 4/211). A total of 73.3% (110/150) of *S. pseudintermedius* isolates were resistant to at least one antimicrobial, with 46% (69/150) MDR and 25.3% (38/150) MRS (Figure 1B).

### 2.4. Methicillin-Resistant Staphylococci

Of the 211 isolates, 57 (27%) were MRS and belonged to 20 (39.2%) of the 51 dogs with staphylococcal isolates. In addition, 38 (18%, 38/211) MRSP isolates were obtained from eleven dogs (21.6%, 11/51), and four methicillin-resistant *Staphylococcus* aureus (MRSA) (1.9%, 4/211) were obtained from three dogs (5.9%).

Of the 57 strains identified as MRS, 48 (84.2%) were positive for the mecA gene and nine (15.8%) lacked the mecA gene but were phenotypically resistant to cefoxitin. The 48 (22.7%) isolates of mecA+ belonged to 17 dogs (33.3%—17/51). Five different species were positive for mecA, but this gene was mostly identified in *S. pseudintermedius* (79.2%). In fact, an *S. pseudintermedius* isolate was 22.2 times more likely to be positive for mecA than the other species (*p* < 0.001). The frequency of MRSP was also evaluated at the three sampling sites (nasal: 16/85—18.9%; rectal: 15/72—20.8%, and axillary: 7/54—13%), and all were found to be statistically similar (*p* = 0.511).

Forty of 48 (83.3%) MRS strains were phenotypically resistant to oxacillin/cefoxitin, which is considered an SCCmec predictor test for staphylococci. With the exception of MRSP, all remaining staphylococcal species that were mecA positive by PCR were susceptible to both oxacillin/cefoxitin, which was inconsistent with the genotypic test (Supplementary File S1). These isolates were considered MRS based on the confirmation of the mecA gene. In addition, nine (4.3%, 9/211) isolates from seven (13%, 7/54) dogs, including four *S. aureus* isolates, showed an antimicrobial resistance profile consistent with MRS (resistance to cefoxitin, penicillin, and at least two other classes of antimicrobials), even though they were not positive for the mecA gene (Supplementary File S1).

### 2.5. Dynamics of Colonization of MRSP in Dogs Admitted to the ICU

**Figure 2:**
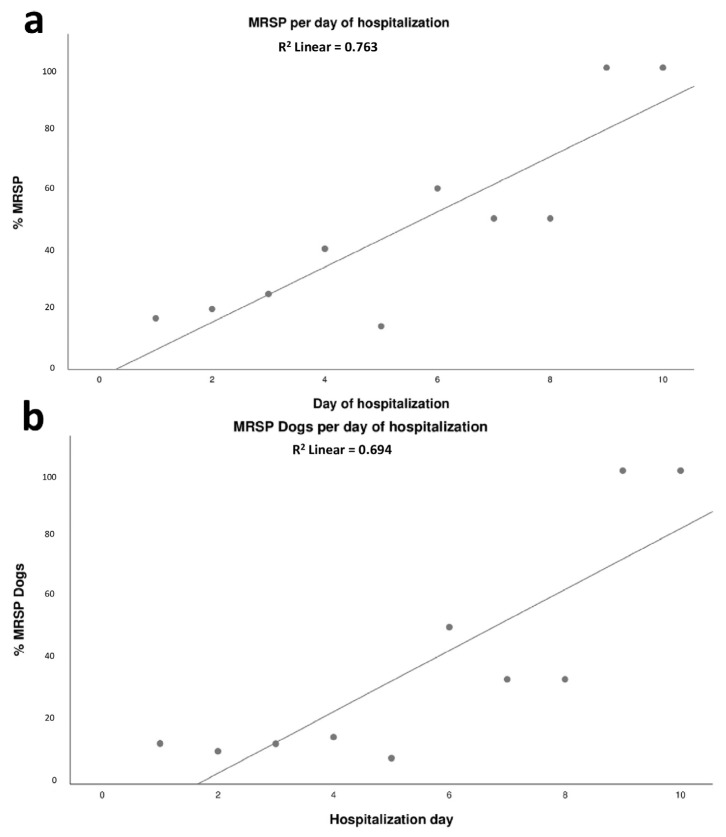
Relationship between the day of hospitalization and the proportion of MRSP strains (**a**); relationship between the day of hospitalization and proportion of dogs positive for MRSP strains (**b**).

A positive association was observed between the length of stay in the ICU (in days) and the proportion of MRSP isolates and dogs positive for MRSP isolation (*p* = 0.001, R^2^ = 0.733). For each day of hospitalization, a 9.1% increase in the total amount of MRSP isolates was observed (Figure 2a); there was also a significant increase in the proportion of MRSP-positive animals (*p* = 0.003, R^2^ = 0.656). In addition, for each day of hospitalization in the ICU a 9.8% increase in the number of MRSP-positive dogs was observed (Figure 2b).

No difference was found in the length of stay between dogs that carried MRSP or MSSP on the first day of hospitalization or in dogs that acquired MRSP after the second day of hospitalization. However, dogs with MRSP strains resistant to nine or more antimicrobial classes (18 of them with identical phenotypic resistance profile) were hospitalized for an average 6.4 days, while the overall mean for all dogs with other MRSP strains was 2.4 days (*p* = 0.009). Eight of these isolates were sequenced and all were identified as ST71, but they were not clonal (Figure 2).

### 2.6. Predictors for MRSP in Dogs Admitted to the ICU of HV-UFMG

Clinical data were evaluated to identify the possible risk factors for the acquisition of MRSP strains in the ICU (Table 4). The risk of colonization by MRSP was higher in female dogs (*p* < 0.001; OR = 7.31), elderly dogs (*p* = 0.016; OR = 2.64), and dogs that had prior antimicrobial use (*p* = 0.017; OR = 2.53).

### 2.7. Comparison of MRSP Isolates from Infected and Colonized Dogs

**Figure 3:**
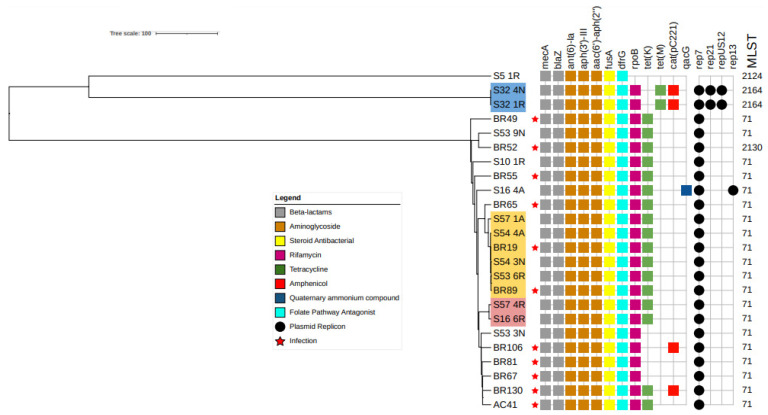
Genetic relationship between MRSP strains isolated from dogs admitted to the ICU of HV-UFMG and *Staphylococcus* spp. infected dogs attended at HV-UFMG [15], between 2018 and 2019. Each isolate was subjected to single nucleotide polimorphism (SNP) analysis using CSIPhylogeny [16] with *S. pseudintermedius* strain DG072 (accession number GCA_016455165.1) as a reference. A minimal Z-score of 1.96 and a minimal depth at SNP position of 10× were used. The percentage of reference genomes covered by all isolates was 81.1%. The tree image was generated using iTOL [17] online using midpoint rooting.

Thirteen isolates colonizing seven dogs from the ICU were subjected to WGS. SCCmec 3A was identified in the seven MRSP isolates where the mobile genetic element was typeable. Unfortunately, in the remaining six isolates ccr was incomplete, making typing impossible. MLST revealed two sequence types: ST71 in most samples, and ST2124 at two sites from one animal (Figure 3). Furthermore, wgMLST and SNP analyses revealed three clones (fewer than 10 alleles in wgMLST and 10 SNPs). The same clone (Figure 3, blue highlight) was isolated from the rectum and nostrils of dog S32 on days one and four, respectively. The second clone (Figure 3, pink highlight) was identified in the rectum of two different dogs, S16 and S57, on the sixth and fourth days in the ICU. Finally, the third clone (Figure 3, yellow highlight) was identified in three dogs: S57 (axilla, first day), S54 (nostril, third day, and axilla, fourth day), and S53 (rectum, third day). These animals were admitted to the ICU on very close days of the experiment (days 234–260).

Eleven isolates from MRSP-infected dogs belonging to the study conducted by Viegas et al. [15] were also sequenced for comparison. Interestingly, the wgMLST and SNP analyses confirmed that the two infections (BR19 and BR89) were caused by the same clone that colonized S57, S54, and S53 (Figure 3, yellow highlight). These dogs were hospitalized or treated in the same institution between days 234 and 260 of the experiment, but were never admitted to the ICU.

## 3. Discussion

During the study, 82% of the dogs admitted to the ICU were colonized by *S. pseudintermedius* and 15.6% by MRSP (Table 1, day 1). The occurrence of *S. pseudintermedius* in dogs is not surprising and is consistent with the current literature [10,18,19,20]. In fact, this agent was recovered from four out of the five dogs sampled, being at least six times more likely to be isolated than any other species identified in this study. On the other hand, the frequency of MRSP-positive dogs was higher than that in previous studies with dogs sampled either in the community or in veterinary hospital care (up to 4.6%) [21,22,23], but similar to that in a previous study in dogs admitted to an ICU [24]. This higher frequency of MRSP-positive dogs in the ICU clearly indicates that this unit as a risk factor for the selection and isolation of MRSP.

The present study also suggested that sampling the nasal and rectal sites presented a higher probability of isolating *S. pseudintermedius* than the axillary site, similar to previous works [18,25]. The detection of *S. aureus* was also higher at the nasal site, showing an almost six-fold greater chance of isolation at this site than for the sum of the other staphylococci. Together, these results suggest that nasal and rectal sites are of great relevance for studies on the dynamics of colonization by *S. pseudintermedius* and *S. aureus* in dogs [18,21,25]. Interestingly, the axillary site showed a higher diversity of staphylococci. The main hypothesis for this is that the axilla easily becomes contaminated by contact with the external environment, including in the handling of the animal by the ICU crew [26]. Despite *S. pseudintermedius*, the vast majority of staphylococci species isolates in the axilla are commonly reported as colonizers of humans [27], which reinforces the hypothesis of contamination and also demonstrates, together with the lower isolation rate, that the axillary site is not preferable for colonization studies in dogs.

Almost half of the isolates in the present study were MDR, including 27% MRS isolates. Reference [24] also reported a similar rate (58.2%) in a study of dogs in the ICU. The high rate of antimicrobial resistance identified might be linked to the extensive antimicrobial use typical in an ICU, which makes this setting extremely suitable for the selection of multidrug-resistant microorganisms [9,28]. A recent systematic review showed that *Staphylococcus* species, mostly MRSP, are among the most prevalent bacteria in HAIs in veterinary institutions [2].

Among all MRS isolates, 18% were identified as MRSP, both for the presence of the *mecA* gene and for oxacillin resistance, in accordance with [29]. On the other hand, all MRSA isolates showed negative results for the *mecA* gene, but resistance to cefoxitin, penicillin, and at least two other non-beta-lactam antimicrobials were designated as phenotypically methicillin-resistant samples [29]. In this context, it is important to note that although the *mecA* gene has not been detected in these strains, it is possible that another resistance determinant from this gene family is involved. Recently, Adiguzel et al. [30] also reported the absence of known *mec* genes in MRS isolates from dogs by PCR, suggesting the need for further studies to better understand this possible alternative source of resistance. The possible circulation of MRSA in the ICU at HV-UFMG is noteworthy given the risk posed by this pathogen to humans [9].

In this study, female sex, old dogs (>7 years), and the previous use of antimicrobials were identified as risk factors for the acquisition of MRSP in the ICU. Previous studies identified prior antimicrobial use as a risk factor for MRSP colonization [9,15,31]. This finding underlines the importance of the prudent use of antimicrobials in pet animals, including hospitalized patients. The greater probability of colonization in female and elderly dogs has not been previously described as a risk factor for MRSP acquisition, and these findings raised some hypotheses. The likelihood of a dog being treated with antimicrobials naturally increases with age. In addition, owing to immunosenescence, elderly dogs are more prone to develop infectious diseases, which would also require antimicrobial treatment and eventually even hospital admissions [32]. It is also possible that females are more susceptible than males due to physiological issues associated with the estrous cycle, since in the progesteronic phase, as well as during pregnancies, females are more susceptible to infection and colonization by microorganisms due to reduced leukocyte activity and consequent immunosuppression [33,34].

In this study, each day of hospitalization in the ICU increased the risk of a dog being colonized by MRSP by almost 10%. Similarly, Gronthal et al. [6], studying an outbreak of MRSP in a Finnish Veterinary Hospital, also reported that prolonged hospital stay increased the probability of acquiring MRSP. In this context, ICU is one of the most important environmental sources of bacteria associated with HAIs in veterinary hospitals [2]. In addition, animals colonized by MRSP isolates resistant to nine or more antimicrobials had a prolonged ICU stay compared to dogs that were positive for MRSP strains resistant to a lower number of antimicrobials. These findings reinforce the need for actions focusing on the control of MRSP in the ICU, including antimicrobial stewardship programs, as this pathogen can directly affect the length of hospital stay, reflecting hospitalization costs and increasing the risk of HAI in these animals.

The majority of the isolates subjected to WGS were identified as ST71, a well-known sequence type frequently reported to be resistant to all antimicrobials commonly used in routine small animal care [35,36,37] and also reported to infect humans [13]. Interestingly, this sequence type was recently reported to infect dogs at the same institution [15]. This recent study demonstrated that isolates recovered from infected surgical wounds acquired in the hospital were more likely to be positive for MRSP with a more complex resistance profile than isolates from other sites [15]. The present study compared these strains with those isolated in the ICU, and revealed that at least one MRSP clone was circulating in the ICU and caused infections in animals housed in other hospital settings at the same institution during the same period of time. This finding indicates that the transmission of this pathogen occurs within the ICU as well as between the ICU and other hospital settings. However, the role of environmental transmission was not addressed in the current study. Recent studies have suggested that poor infection prevention and control standards are associated with environmental contamination by MDR pathogens, including MRSP [38]. Thus, our current findings, together with recently published reports, clearly emphasize the need for surveillance efforts and proper infection control strategies in institutions of this kind. 

Another MRSP clone was confirmed to be circulating in the ICU once it was detected in two animals at different time points. Interestingly, some recent studies have shown that MRSP isolates containing SCC*mec* 3A tend to be healthcare-related strains, including in humans [38,39,40,41,42,43], which is in agreement with the results of the present study where all isolates with the SCC*mec* type were classified as 3A. 

Our study suggests that MRSP acquisition can affect the length of stay in an ICU, directly affecting the costs and beds available. In addition, the detection of the same MRSP clone colonizing animals in the ICU and causing HAI in other hospital settings clearly calls for a review of the infection prevention and control practices at the institution. In this context, the risk factors for MRSP colonization in the ICU revealed in the present study can be used to guide surveillance programs and further actions to control MRSP colonization and infection.

## 4. Materials and Methods

### 4.1. Strain Collection, Isolation, and Identification

This longitudinal study was conducted between August 2018 and April 2019 (264 days). A total of 54 dogs admitted to the ICU of the Veterinary Hospital of the Federal University of Minas Gerais (HV-UFMG) were included in the study. After obtaining informed consent from the owners, each dog included in the study underwent the collection of sterile swabs from three distinct body sites (nostril, axilla, and rectum), once a day, from the first to the last day of hospitalization or death. Epidemiological data were obtained by evaluating the participants’ medical records. Information regarding age, sex, breed, size, reason and time of hospitalization, comorbidities, prior use of antimicrobials (two months) or during hospitalization, profession of the owner (health professionals or others), coexistence with other animals, and outcome (death or hospital discharge) was collected.

All the bacterial swabs were plated on a selective medium (Mannitol salt agar, Kasvi, Mumbai, India ) and incubated for 18 to 24 h at 37 °C. Up to two colonies from each sample were plated on Müller Hinton agar (Difco, Sparks, Maryland, USA) and identified using matrix-assisted laser desorption/ionization time-of-flight (MALDI-TOF) mass spectrometry, as described previously [44], and using a FlexControl MicroFlex LT mass spectrometer (Bruker Daltonics, Billerica, Massachusetts, EUA). Prior to the measurements, calibration was performed using standard control (*Escherichia coli* DH5 alpha; Bruker Daltonics, Billerica, Massachusetts, EUA). The real-time (RT) identification score criteria used were those recommended by the manufacturer: a score ≥ 2.3 indicated a species-level identification. Isolates identified as *Staphylococcus intermedius* group (SIG) by MALDI-ToF were confirmed by multiplex polymerase chain reaction (PCR) of the *nuc* gene [45]. Non-SIG isolates with a MALDI-TOF score > 2.3 were submitted for the sequencing of the *rpoB* gene, as previously described [46].

### 4.2. Antimicrobial Susceptibility Testing

All isolates were tested using the disk diffusion method, and the zone of inhibition for each antimicrobial was interpreted according to the Clinical and Laboratory Standards Institute (CLSI) documents, M100-Ed31 [29], and VET01S-Ed5 [47]. The antimicrobials tested in the present study were selected based on a previous work [15] and aiming to cover the most frequent antimicrobial classes used in veterinary medicine. The following antimicrobials were tested: oxacillin (OXA, 1 µg), cefoxitin (FOX, 30 µg), penicillin (PEN, 10 IU), gentamicin (GEN, 10 µg), erythromycin (ERY, 15 µg), clindamycin (CLI, 2 µg), tetracycline (TET, 30 µg), ciprofloxacin (CIP, 5 µg), nitrofurantoin (NIT, 300 µg), trimethoprim-sulfamethoxazole (SXT, 1.25/23.75 µg), chloramphenicol (CHL, 30 µg), enrofloxacin (5 µg), and rifampicin (RIF, 5 µg) (Oxoid, Basingstoke, United Kingdom). *Staphylococcus aureus* ATCC^®^ 25923 was used as the control. Strains were considered multidrug resistant (MDR) when they were resistant to three or more classes of antimicrobials [48].

### 4.3. DNA Extraction and Detection of mecA

The extraction of bacterial genomic DNA with guanidium thiocyanate was performed as previously described [49]. The extracted DNA was quantified using a NanoDrop spectrophotometer (Thermo Fisher Scientific, Wilmington, DE, USA). The purity of the extracted DNA was determined by measuring the absorbance ratio at 260/280 nm. All staphylococci isolates were tested by PCR to determine whether they carried the mecA gene [50].

### 4.4. Whole-Genome Sequencing

A total of 24 *S. pseudintermedius* isolates were subjected to whole-genome sequencing. Thirteen MRSP strains belonged to this study, and eleven belonged to a recent study performed during the same period and at the same veterinary hospital, with clinical samples from MRSP-infected dogs in the same period [15]. These samples were included to evaluate the genetic similarity of isolates obtained from dogs from the ICU, with isolates originating from infected animals admitted to other hospital settings. The genome accession numbers are provided in Supplementary File S1.

The strains were incubated on Mueller–Hinton agar at 37 °C for 24 h. Genomic DNA was extracted using a Maxwell 16^®^ Research Instrument (Promega, Madison, Wisconsin, EUA) combined with lysozyme and proteinase K (10 mg/mL and 20 mg/Ml, respectively). Genome sequencing was performed using the Illumina NextSeq platform (mid-out 2 × 150 bp cycles), and the raw data were analyzed using FastQC (Babraham Bioinformatics, Cambridge, England). The assembly was performed using SPAdes 3.5.0 [51]. ResFinder 4.1 [52,53,54] and PlasmidFinder 2.1 [52,55] were used to identify acquired antimicrobial resistance determinants and conjugative plasmid replicons, respectively. SCCmecFinder 1.2 was used for SCCmec typing [39,56]. MLST 2.0 was used to determine sequencing types [52,57,58,59] according to the Perreten scheme [60]. The raw reads of each isolate were subjected to SNP analysis using CSIPhylogeny [16], with *S. pseudintermedius* strain DG072 (accession number GCA_016455165.1) as a reference. The tree image was generated using ITOL online using midpoint rooting [17].

### 4.5. Statistical Methods

The Chi-square test of adherence was used to assess the distribution of the data. The contagion behavior in relation to the factors of interest was assessed using contingency tables. The association between categorical variables and isolated strains was evaluated by a univariate analysis using Fisher’s exact test. Associations were expressed as odds ratios (ORs) and 95% confidence intervals (CIs), and the statistical significance was set at *p* ≤ 0.05. To perform the statistical analyses for age, the animals were categorized into scores according to Harvey et al. [61]: 1 for puppies (≤12 months), 2 for adults (>12 and ≤84 months), and 3 for the elderly (≥84 months). For quantitative variables, the Mann–Whitney test was used with a *p* ≤ 0.05, and Tukey’s comparison test was applied if statistical significance was observed. Linear regression was applied to assess the relationship between the length of hospitalization and factors of interest. All analyses were performed using R Software 4.0.9 (R Development Core Team, NZ).

## Figures and Tables

**Table 1:** Clinical, epidemiological, and laboratory variables of dogs admitted to the Intensive Care Unit of the Veterinary Hospital of the Federal University of Minas Gerais.

Variable	Reference	Value
Sex (*n* = 53)	Male	19 (35.8%)
	Female	34 (64.2%)
Dog size (*n* = 53)	Large breed	17 (32.1%)
	Medium breed	20 (37.7%)
	Small breed	16 (30.2%)
Household with other animals (*n* = 43)	Yes	30 (69.8%)
	No	13 (30.2%)
Comorbidity (*n* = 53)	Yes	25 (47.2%)
	No	28 (52.8%)
Age (months; *n* = 46) ^1^	Puppies (<12)	6 (13%)
	Adults (≥12 and <84)	17 (37%)
	Elderly (≥84)	23 (50%)
Length of ICU stay (*n* = 54)	1 to 2 days	23 (42.6%)
	3 to 4 days	18 (33.3%)
	>4 days	13 (24.1%)
Previous use of antimicrobial (*n* = 41)	Yes	20 (48.8%)
	No	21 (51.2%)
Antimicrobial use during ICU stay (*n* = 53)	Yes	41 (77.4%)
	No	12 (22.6%)
Clinical outcome (*n* = 53)	Release from ICU	33 (62.3%)
	Death	20 (37.7%)

^1^ Puppies (<12 monts), Adults (≥12 and <84 months), Elderly (≥84 months).

**Table 2:** Frequency of total and per day hospitalization isolates of *Staphylococcus* spp., *S. pseudintermedius* and MRSP recovered from 54 dogs admitted to the ICU of HV-UFMG between August 2018 and April 2019.

Day of Hospitalization	*Staphylococcus* sp.		*S. pseudintermedius*		MRSP	
	Isolates (%)	Dogs (%)	Isolates (%)	Dogs (%)	Isolates (%)	Dogs (%)
1	97/162 (59.9)	45/54 (83.3)	65/97 (67)	37/45 (82.2)	11/97 (11.3)	7/45 (15.6)
2	37/114 (32.5)	23/38 (60.5)	25/37 (67.6)	19/23 (82.6)	5/37 (13.5)	4/23 (17.4)
3	29/93 (31.2)	19/31 (61.3)	20/29 (69)	15/19 (78.9)	5/29 (17.2)	4/19 (21)
4	25/60 (41.7)	14/20 (70)	20/25 (80)	11/14 (78.6)	8/14 (32)	3/14 (21.4)
5	8/36 (22.2)	6/12 (50)	7/8 (87.5)	5/6 (83.3)	1/8 (12)	1/6 (16.7)
6	6/12 (50)	4/4 (100)	5/6 (83.3)	4/4 (100)	3/6 (50)	2/4 (50)
7	2/9 (22.2)	2/3 (66.7)	2/2 (100)	2/2 (100)	1/2 (50)	1/2 (50)
8	5/9 (55.6)	3/3 (100)	4/5 (80)	3/3 (100)	2/5 (40)	1/3 (33.3)
9	1/3 (33.3)	1/1 (100)	1/1 (100)	1/1 (100)	1/1 (100)	1/1 (100)
10	1/3 (33.3)	1/1 (100)	1/1 (100)	1/1 (100)	1/1 (100)	1/1 (100)
Total	211/501 (42.1)	51/54 (94.4)	150/211 (71)	45/51 (88.2)	38/211 (18)	11/51 (21.6)

**Table 3:** Species distribution and frequency of isolates (total and per collection site).

*Staphylococcus* sp.	Isolates	Animals	Nasal Isolates	Rectal Isolates	Axillary Isolates
*S. pseudintermedius*	150/211 (71.1%)	45/51 (88.2%)	59/150 (39.3%) ^d^	57/150 (38%) ^d^	34/150 (22.7%) ^e^
*S. aureus*	27/211 (12.8%)	13/51 (25.5%)	19/27 (70.4%) ^f^	2/27 (7.4%) ^g^	6/27 (22.2%) ^g^
*S. haemolyticus*	13/211 (6.2%)	9/51 (17.6%)	5/13 (38.5%)	4/13 (30.8%)	4/13 (30.8%)
*S. devriese*	4/211 (1.9%)	2/51 (3.9%)	0/4 (0%)	1/4 (25%)	3/4 (75%)
*S. felis*	3/211 (1.4%)	3/51 (5.9%)	0/3 (0%)	3/3 (100%)	0/3 (0%)
*S. epidermidis*	3/211 (1.4%)	2/51 (3.9%)	0/3 (0%)	1/3 (33.3%)	2/3 (66.7%)
*S. hominis*	3/211 (1.4%)	3/51 (5.9%)	1/3 (33.3%)	1/3 (33.3%)	1/3 (33.3%)
*S. simulans*	3/211 (1.4%)	3/51 (5.9%)	0/3 (0%)	2/3 (66.7%)	1/3 (33.3%)
*S. saprophyticus*	2/211 (1.0%)	2/51 (3.9%)	1/2 (50%)	0/2 (0%)	1/2 (50%)
*S. delphini*	1/211 (0.5%)	1/51 (1.9%)	0/1 (0%)	0/1 (0%)	1/1 (100%)
*S. schleiferi*	1/211 (0.5%)	1/51 (1.9%)	0/1 (0%)	1/1 (100%)	0/1 (0%)
*S. equorum*	1/211 (0.5%)	1/51 (1.9%)	0/1 (0%)	0/1 (0%)	1/1 (100%)
Total **	211 (100%)	51 (100%)	85/211 (50.9%) ^a^	72/211 (43.1%) ^ab^	54/211 (32.3%) ^b^

The recently approved differentiation of *S. schleiferi* into two species (*S. coagulans* and *S. schleiferi*) was not considered in this study. ** Similar letters in the rows indicate that there was no statistical difference. Different letters indicate statistically significant differences at the 5% level (*p* < 0.05, Chi-square test).

**Table 4:** Variables evaluated as possible risk factors associated with MRSP in the ICU of HV-UFMG between August 2018 and April 2019.

Variable	MRSP Isolates	*p*-Value	OR
Sex			
Male	4/84 (5%)	<0.001	7.31
Female	34/127 (27%)		
Size			
Small (S)	14/79 (18%)	S × M = 0.52; S × L = 0.66; M × L = 0.18	NA
Medium (M)	14/60 (23%)		
Large (L)	10/72 (14%)		
Age			
Young (≤12 months) (Y)	4/24 (17%)	(Y + A) × E = 0.016	2.64
Adult (>12 e ≥ 84 months) (A)	7/56 (13%)		
Elderly (>84 months) (E)	27/91 (30%)		
Contact with other animals			
Yes	31/119 (26%)	0.27	NA
No	6/37 (16%)		
Comorbidity			
Yes	22/123 (18%)	1.0	NA
No	16/88 (18%)		
Prior use of antimicrobials			
Yes	24/74 (32%)	0.017	2.53
No	11/69 (16%)		
Antimicrobial use during hospitalization			
Yes	26/155 (17%)	0.43	NA
No	12/56 (21%)		
Beta-lactams use during hospitalization			
Yes	26/129 (20%)	0.71	NA
No	12/71 (17%)		
Death			
Yes	1/20 (5%)	0.14	NA
No	37/191 (19%)		

## Data Availability

Part of the data presented in this study are openly available in GenBank and the genome accession numbers of all genomes used can be found in the Table S6. The remaining data presented in this study are available on request from the corresponding author.

## Supplementary Materials

The following supporting information can be downloaded at: https://www.mdpi.com/article/10.3390/antibiotics12030621/s1, Table S1: Details of the antimicrobial resistance among staphylococci isolated from dogs admitted to the ICU of HV-UFMG in Belo Horizonte, Minas Gerais, between August 2018 and April 2019; Table S2: Antimicrobial resistance among *Staphylococcus aureus*, *S. pseudintermedius* and other staphylococci isolated from dogs admitted to the ICU of HV-UFMG in Belo Horizonte, Minas Gerais, between August 2018 and April 2019; Table S3: Total isolates, frequency, and staphylococcal species that had the *mecA* gene; Table S4: Staphylococcal species and total isolates that showed phenotypic methicillin resistance profile (MRS); Table S5: Breakpoints used to determine the breakpoints for oxacillin and cefotaxime for Staphylococcal species; Table S6: Isolates and accession number of methicillin-resistant *Staphylococcus pseudintermedius* (MRSP) isolated from nostril, axilla, and rectum from dogs admitted to the Intensive Care Unit of the Veterinary Hospital of the Federal University of Minas Gerais and from dogs diagnosed with MRSP infection in the same institution.
