# Supplementary material for: Risk Factors, Genetic Diversity, and Antimicrobial Resistance of Staphylococcus spp. Isolates in Dogs Admitted to an Intensive Care Unit of a Veterinary Hospital
Source: Antibiotics (Basel). 2023 Mar 21;12(3):621. doi: 10.3390/antibiotics12030621 (PMC10045350; doi:10.3390/antibiotics12030621)
Supplement: Supplementary file 1 [file antibiotics-12-00621-s001.zip › antibiotics-2275776-supplementary.pdf]

Supplementar Material

Supplementary Table S1 – Details of the antimicrobial resistance among staphylococci isolated from dogs admitted to the ICU of HV-UFMG in Belo Horizonte, Minas Gerais, between August 2018 and April 2019.

Species	Isolates	OXA/FOX	PEN	TET	SXT	ERI	CLI	RIF	CIP	GEN	ENO	CHL	NIT	MRS	MDR
**SIG**	**151 (71.5%)**	**38 (18%)**	**97 (46%)**	**88 (41.7%)**	**75 (35.5%)**	**52 (24.6%)**	**52 (24.6%)**	**44 (20.8%)**	**42 (19.9%)**	**42 (19.9%)**	**41 (19.4%)**	**13 (6.2%)**	**1 (0.5%)**	**38 (18.0%)**	**69 (32.7%)**
S. pseudintermedius	150 (71.1%)	38 (18.0%)	96 (45.5%)	87 (41.2%)	75 (35.5%)	52 (24.6%)	52 (24.6%)	44 (20.8%)	42 (19.9%)	42 (19.9%)	41 (19.4%)	13 (6.2%)	1 (0.5%)	38 (18.0%)	69 (32.7%)
S. delphini	1 (0.5%)	0 (0.0%)	1 (0.5%)	1 (0.5%)	0 (0.0%)	0 (0.0%)	0 (0.0%)	0 (0.0%)	0 (0.0%)	0 (0.0%)	0 (0.0%)	0 (0.0%)	0 (0.0%)	0 (0.0%)	0 (0.0%)
**Non-SIG**	**60 (28.4%)**	**14 (6.6%)**	**25 (11.8%)**	**22 (10.4%)**	**19 (9.0%)**	**24 (11.4%)**	**22 (10.4%)**	**6 (2.8%)**	**15 (7.1%)**	**10 (4.7%)**	**15 (7.1%)**	**3 (1.4%)**	**3 (1.4%)**	**19 (9.0%)**	**23 (10.9%)**
S. aureus	27 (12.8%)	4 (1.9%)	11 (5.2%)	10 (4.7%)	7 (3.3%)	9 (4.3%)	8 (3.8%)	3 (1.4%)	6 (2.8%)	5 (2.4%)	6 (2.8%)	2 (0.9%)	2 (0.9%)	4 (1.9%)	8 (3.8%)
S. haemolyticus	13 (6.2%)	2 (0.9%)	6 (2.8%)	5 (2.4%)	2 (0.9%)	6 (2.8%)	3 (1.4%)	1 (0.5%)	2 (0.9%)	1 (0.5%)	2 (0.9%)	0 (0.0%)	0 (0.0%)	7 (3.3%)	3 (1.4%)
S. devriese	4 (1.9%)	0 (0.0%)	2 (0.9%)	0 (0.0%)	0 (0.0%)	2 (0.9%)	2 (0.9%)	0 (0.0%)	0 (0.0%)	0 (0.0%)	0 (0.0%)	0 (0.0%)	0 (0.0%)	1 (0.5%)	2 (0.9%)
S. felis	3 (1.4%)	3 (1.4%)	2 (0.9%)	2 (0.9%)	2 (0.9%)	2 (0.9%)	2 (0.9%)	1 (0.5%)	2 (0.9%)	2 (0.9%)	2 (0.9%)	0 (0.0%)	0 (0.0%)	2 (0.9%)	3 (1.4%)
S. epidermidis	3 (1.4%)	2 (0.9%)	0 (0.0%)	2 (0.9%)	3 (1.4%)	0 (0.0%)	0 (0.0%)	0 (0.0%)	2 (0.9%)	0 (0.0%)	2 (0.9%)	0 (0.0%)	0 (0.0%)	1 (0.5%)	2 (0.9%)
S. hominis	3 (1.4%)	1 (0.5%)	2 (0.9%)	1 (0.5%)	2 (0.9%)	3 (1.4%)	2 (0.9%)	1 (0.5%)	1 (0.5%)	1 (0.5%)	1 (0.5%)	1 (0.5%)	1 (0.5%)	2 (0.9%)	2 (0.9%)
S. simulans	3	1	1	1	1	1	3	0	1	0	1	0	0	1	2

Supplementar Material

	(1.4%)	(0.5%)	(0.5%)	(0.5%)	(0.5%)	(0.5%)	(1.4%)	(0.0%)	(0.5%)	(0.0%)	(0.5%)	(0.0%)	(0.0%)	(0.5%)	(0.9%)
S. saprophyticus	2 (1.0%)	1 (0.5%)	1 (0.5%)	1 (0.5%)	1 (0.5%)	1 (0.5%)	1 (0.5%)	0 (0%)	1 (0.5%)	1 (0.5%)	1 (0.5%)	0 (0%)	0 (0%)	1 (0.5%)	1 (0.5%)
S. schleiferi*	1 (0.5%)	0 (0.0%)	0 (0.0%)	0 (0.0%)	1 (0.5%)	0 (0.0%)	0 (0.0%)	0 (0.0%)	0 (0.0%)	0 (0.0%)	0 (0.0%)	0 (0.0%)	0 (0.0%)	0 (0.0%)	0 (0.0%)
S. equorum	1 (0.5%)	0 (0.0%)	0 (0.0%)	0 (0.0%)	0 (0.0%)	0 (0.0%)	1 (0.5%)	0 (0.0%)	0 (0.0%)	0 (0.0%)	0 (0.0%)	0 (0.0%)	0 (0.0%)	0 (0.0%)	0 (0.0%)
**Total**	**211 (100%)**	**52^{c} (24.6%)**	**122^{f} (57.8%)**	**110^{ef} (52.1%)**	**94de (44.5%)**	**76^{d} (36.0%)**	**74^{d} (35.0%)**	**50^{c} (23.7%)**	**57^{c} (27.0%)**	**52^{c} (24.6%)**	**56^{c} (26.5%)**	**16^{b} (7.6%)**	**4^{a} (1.9%)**	**57 (27.0%)**	**92 (43.6%)**

Supplementar Material

Supplementar Table S2 - Antimicrobial resistance among *Staphylococcus aureus*, *S. pseudintermedius* and other staphylococci isolated from dogs admitted to the ICU of HV-UFMG in Belo Horizonte, Minas Gerais, between August 2018 and April 2019.

Species	Isolates	OXA/FOX	PEN	TET	SXT	ERY	CLI	RIF	CIP	GEN	ENO	CHL	NIT	MRS	MDR
S. pseudintermedius	150 (100%)	38 (25.3%)	96 (64%)	87 (58%)	75 (50%)	52 (34.7%)	52 (34.7%)	44 (29.3%)	42 (28%)	42 (28%)	41 (27.3%)	13 (8.7%)	1 (0.7%)	38 (25.3%)	69 (46%)
S. aureus	27 (100%)	4 (14.8%)	11 (40.7%)	10 (37%)	7 (25.9%)	9 (33.3%)	8 (29.6%)	3 (11.1%)	6 (22.2%)	5 (18.5%)	6 (22.2%)	2 (7.4%)	2 (7.4%)	4 (14.8%)	8 (29.6%)
Other staphylococci species[1]	34 (100%)	10 (29.4%)	15 (44.1%)	13 (38.2%)	12 (35.3%)	15 (44.1%)	14 (41.2%)	3 (8.8%)	9 (26.5%)	5 (14.7%)	9 (26.5%)	1 (2.9%)	1 (2.9%)	5 (14.7%)	15 (44.1%)
**Total**	**211 (100%)**	**52^{c} (24.6%)**	**122^{f} (57.8%)**	**110^{ef} (52.1%)**	**94de (44.5%)**	**76^{d} (36%)**	**74^{d} (35%)**	**50^{c} (23.7%)**	**57^{c} (27%)**	**52^{c} (24.6%)**	**56^{c} (26.5%)**	**16^{b} (7.6%)**	**4^{a} (1.9%)**	**57 (27%)**	**92 (43.6%)**

Supplementary Table S3 - Total isolates, frequency, and staphylococcal species that had the *mecA* gene.

Species	Frequency among *mecA*$^{+}$ isolates	*mecA*+ but CEF/OXA susceptibility
S. pseudintermedius	38/48 (79.2%)	0/38 (0.0%)
S. haemolyticus	7/48 (14.6%)	5/7 (71.4%)
S. hominis	1/48 (2.1%)	1/1 (100.0%)
S. epidermidis	1/48 (2.1%)	1/1 (100.0%)
S. devriesei	1/48 (2.1%)	1/1 (100.0%)
**Total**	**48/48 (100.0%)**	**8/48 (16.7%)**

Supplementary Table S4 - Staphylococcal species and total isolates that showed phenotypic methicillin resistance profile (MRS).

Supplementar Material

Species	Frequency (%)
S. aureus	4/9 (44.4%)
S. felis	2/9 (14.6%)
S. hominis	1/9 (11.1%)
S. simulans	1/9 (11.1%)
S. saprophyticus	1/9 (11.1%)
**Total**	**9/48 (100.0%)**

Supplementary Table S5 – Breakpoints used to determine the breakpoints for oxacillin and cefotaxime for Staphylococcal species.

	**Antimicrobial Zone Diameter Breakpoints**	
***Staphylococcus* species**	**Oxacillin**	**Cefoxitin**
S. pseudintermedius and *S. schleiferi*	R: ≤17 mm /S: ≥18 mm	NA
S. aureus and *S. lugdunensis*	NA	R: ≤21 mm /S: ≥22 mm
Other *Staphylococcus* species	NA	R: ≤24 mm /S: ≥25 mm

Supplementar Material

Supplementary Table S6: Isolates and accession number of methicillin-resistant *Staphylococcus pseudintermedius* (MRSP) isolated from nostril, axilla, and rectum from dogs admitted to the Intensive Care Unit of the Veterinary Hospital of the Federal University of Minas Gerais and from dogs diagnosed with MRSP infection in the same institution.

Isolate	Source (Body site)	Genome Accession	Reference
**S5.1R**	Colonization (Rectum)	SAMN32679007	This study
**S32.4N**	Colonization (Nostril)	SAMN32678995	
**S32.1R**	Colonization (Rectum)	SAMN32678987	
**S53.9N**	Colonization (Nostril)	SAMN32679010	
**S10.1R**	Colonization (Rectum)	SAMN32679008	
**S16.4A**	Colonization (Axilla)	SAMN32678988	
**S54.4A**	Colonization (Axilla)	SAMN32679004	
**S54.3N**	Colonization (Nostril)	SAMN32678993	
**S53.6R**	Colonization (Rectum)	SAMN32678992	
**S57.4R**	Colonization (Rectum)	SAMN32679001	
**S17.6R**	Colonization (Rectum)	SAMN32679009	
**S53.3N**	Colonization (Nostril)	SAMN32679002	
**BR49**	Infection	SAMN32679003	Vieira et al. (2022)
**BR52**		SAMN32678998	
**BR65**		SAMN32678989	
**BR19**		SAMN32679005	
**BR89**		SAMN32678991	
**BR106**		SAMN32678999	
**BR81**		SAMN32678990	
**BR67**		SAMN32678994	
**BR130**		SAMN32678997	
**AC41**		SAMN32678996	
